# Online Gradient Descent for Kernel-Based Maximum Correntropy Criterion

**DOI:** 10.3390/e21070644

**Published:** 2019-06-29

**Authors:** Baobin Wang, Ting Hu

**Affiliations:** 1 School of Mathematics and Statistics, South-Central University for Nationalities, Wuhan 430074, China; 2 School of Mathematics and Statistics, Wuhan University, Wuhan 430072, China

**Keywords:** correntropy, maximum correntropy criterion, online algorithm, robustness, reproducing kernel Hilbert spaces

## Abstract

In the framework of statistical learning, we study the online gradient descent algorithm generated by the correntropy-induced losses in Reproducing kernel Hilbert spaces (RKHS). As a generalized correlation measurement, correntropy has been widely applied in practice, owing to its prominent merits on robustness. Although the online gradient descent method is an efficient way to deal with the maximum correntropy criterion (MCC) in non-parameter estimation, there has been no consistency in analysis or rigorous error bounds. We provide a theoretical understanding of the online algorithm for MCC, and show that, with a suitable chosen scaling parameter, its convergence rate can be min–max optimal (up to a logarithmic factor) in the regression analysis. Our results show that the scaling parameter plays an essential role in both robustness and consistency.

## 1. Introduction

Regression analysis is an important problem in many fields of science. The traditional least squares method may be the most used algorithm for regression in practice. However, it only relies on the mean squared error and belongs to second-order statistics, whose optimality depends heavily on the assumption of Gaussian noise. Thus, it usually performs poorly when the noise is not normally distributed. Alterative approaches have been proposed to deal with outliers or heavy-tailed distributions. A generalized correlation function named correntropy [1] is introduced as a substitute for the least squares loss, and the maximum correntropy criterion (MCC) [2,3,4,5] is used to improve robustness in situations of non-Gaussian and heavy-tailed error distributions. Recently, MCC has been succeeded in many real applications, e.g., wind power forecasting and pattern recognition [6,7].

In the standard framework of statistical learning, let $X\in R^{n}$ be an explanatory variable with values taken in a compact metric space (X,d),*Y* be a real response variable with Y∈Y⊂R. Here we investigate the application of MCC in the following regression model

$$\begin{array}{c} Y=f_{\rho }\left(X\right)+\epsilon ,\;E\left(\epsilon |X=x\right)=0, \end{array}$$

where ϵ is the noise and $f_{\rho }\left(x\right)$ is the regression function, defined as the conditional mean E(Y|X=x) at each x∈X. The purpose of regression is to estimate the unknown target function $f_{\rho }$ according to the sample $z={\left\{z_{i}=\left(x_{i},y_{i}\right)\right\}}_{i=1}^{T},$ which is drawn independently from the underlying unknown probability distribution ρ on Z:=X×Y. For a hypothesis function f:X→Y, with the scaling parameter σ>0, the correntropy between f(X) and *Y* is defined by $V_{\sigma }\left(f\right):=EG\left(\frac{{\left(f\left(X\right)-Y\right)}^{2}}{2\sigma^{2}}\right)$ where G(u) is the Exponential function $\exp\left\{-u\right\},u\in R.$ For the given sample z, the empirical form of $V_{\sigma }$ is ${\hat{V}}_{\sigma }\left(f\right):=\frac{1}{T}\sum_{i=1}^{T}G\left(\frac{{\left(f\left(x_{i}\right)-y_{i}\right)}^{2}}{2\sigma^{2}}\right).$ When applied to regression problems, MCC intends to maximize the empirical correntropy ${\hat{V}}_{\sigma }$ over a certain underlying hypothesis space H, that is

(1) $$\begin{array}{c} f_{z,H}:=\arg\max_{f\in H}{\hat{V}}_{\sigma }\left(f\right). \end{array}$$

MCC in regression problems has shown its efficiency for cases when the noises are non-Gaussian, and also with large outliers, see [8,9,10]. It also has drawn much attention in the signal processing, machine learning and optimization communities [2,5,11,12,13,14].

Let K:X×X→R be a *Mercer kernel*, i.e., a continuous, symmetric and positive semi-definite function. We say that *K* is a positive semi-definite, if for any finite set $\left\{u_{1},\cdots ,u_{m}\right\}\subset X$ and m∈N, the matrix ${\left(K(u_{i},u_{j})\right)}_{i,j=1}^{m}$ is positive semi-definite. An RKHS $\left(H_{K},∥\cdot {∥}_{K}\right)$ associated with the Mercer kernel *K* is defined as the completion of the linear span of the functions set $\{K_{x}:=K\left(x,\cdot \right),x\in X\}.$ It has the reproducing property

(2) $$\begin{array}{c} f\left(x\right)={\langle f,K_{x}\rangle }_{K} \end{array}$$

for any $f\in H_{K}$ and x∈X. Since X is compact, the RKHS $H_{K}$ is contained in C(X), the space of continuous functions on X with the norm ${∥f∥}_{\infty }:=\underset{x\in X}{\sup}\left|f\left(x\right)\right|.$ Moreover, if X is a Euclidean ball in $R^{n}$ with some $\alpha >\frac{n}{2},$ then the Sobolev space $H^{\alpha }\left(X\right)$ is an RKHS. For more families of RKHS in statistical learning, one can refer to [15]. Denote $\kappa :=\sup_{x\in X}\sqrt{K(x,x),}$ then, by the reproducing property (2), there holds

(3) $$\begin{array}{c} {∥f∥}_{\infty }\leq \kappa {∥f∥}_{K},\;for\;any\;f\in H_{K}. \end{array}$$

Denote $\ell_{\sigma }:R\times R\to R$ as the correntropy induced regression loss, given by

$$\begin{array}{c} \ell_{\sigma }\left(u,v\right):=\sigma^{2}\left(1-G\left(\frac{{\left(u-v\right)}^{2}}{2\sigma^{2}}\right)\right)=\sigma^{2}\left(1-\exp\left\{-\frac{{\left(u-v\right)}^{2}}{2\sigma^{2}}\right\}\right). \end{array}$$

Associated with this regression loss $\ell_{\sigma }$ and the RKHS $H_{K},$ MCC for regression (1) in the context of learning theory is reformulated as

(4) $$\begin{array}{c} f_{z}:=\arg\min_{f\in H_{K}}\frac{1}{T}\sum_{i=1}^{T}\ell_{\sigma }\left(f\left(x_{i}\right),y_{i}\right). \end{array}$$

Notice that $\ell_{\sigma }$ is not convex, MCC algorithms are usually implemented by various gradient descent methods [14,16,17]. In this paper, we take the online gradient descent method as follows to solve the above optimization scheme (4) since it is scalable to large datasets and applicable to situations where the samples are presented in sequence.

**Definition** **1.** *Given the sample $z={\left\{z_{i}=\left(x_{i},y_{i}\right)\right\}}_{i=1}^{T},$ the online gradient descent method for MCC is defined by $f_{1}=0,$ and*

(5) $$\begin{array}{c} f_{t+1}=f_{t}-\eta \ell_{\sigma }^{\prime }\left(f_{t}\left(x_{t}\right),y_{t}\right)K_{x_{t}},\;t\in N, \end{array}$$

*where η>0 is the step size and $\ell_{\sigma }^{\prime }$ denotes the derivative of $\ell_{\sigma }$ with respect to the first variable.*

In the literature, most MCC algorithms have been implemented for linear models and cannot be applied to analysis of data with nonlinear structures. Kernel methods provide efficient non-parametric learning algorithms for dealing with nonlinear features. So, RKHS are used in this work as hypothesis spaces in the design of learning algorithms.

An online algorithm for MCC has been used in practical applications for more than one decade, but there still is a lack of the theoretical guarantee or strict analysis for its asymptotical convergence. Because the optimization problem arising from MCC is not convex, the global optimization convergence of the online algorithm (5) for MCC is not unconditionally guaranteed. This also makes the theoretical analysis for MCC essentially difficult. In fact, vast numerical studies show that MCC can lead robust estimators while keeping convenient convergence properties. Thus, our goal is to fill the gap between the theoretical analysis and the optimization process so that the output function of the online algorithm (5) can converge to a global minima while the existing work can not ensure the global optimization of this output. To this end, we study the approximation ability of $f_{T+1}$ generated by (5) at the *T*-iteration to the regression function $f_{\rho }.$ We derive the explicit error rate for (5) with suitable choice of step sizes, which is competitive with those in the regression analysis. In this work, we show that the scaling parameter σ plays an important role in providing robustness and a fast convergence rate.

## 2. Preliminaries and Main Results

We begin with some preliminaries and notations. Throughout the paper, we assume that the unknown distribution ρ on Z=X×Y can be decomposed into the marginal distribution $\rho_{X}$ on X and the conditional distribution ρ(·|x) at each x∈X. We also require that |Y|<M almost surely for some M>1. In the regression analysis, the approximation power of $f_{T+1}$ by (5) is usually measured in terms of the mean squared error in $L_{\rho_{X}}^{2}$-metric $∥f_{T+1}-f_{\rho }{∥}_{\rho },$ that is defined as ${∥\cdot ∥}_{\rho }={∥\cdot ∥}_{L_{\rho_{X}}^{2}}:={\left(\int_{X}{\left|\cdot \right|}^{2}d\rho_{X}\right)}^{\frac{1}{2}}.$

To present our main result for the error bound of $f_{T+1}-f_{\rho }$, the assumption on the target function $f_{\rho }$ will be given as below. Define an integral operator $L_{K}:L_{\rho_{X}}^{2}⟶L_{\rho_{X}}^{2}$ associated with the kernel *K* by

$$\begin{array}{c} L_{K}\left(f\right):=\int_{X}f\left(x\right)K_{x}d\rho_{X},\;f\in L_{\rho_{X}}^{2}. \end{array}$$

By the reproducing property (2) of $H_{K},$ for any $f\in H_{K},$ it can be expressed as

(6) $$\begin{array}{c} L_{K}\left(f\right)=\int_{X}{\langle f,K_{x}\rangle }_{K}K_{x}d\rho_{X}. \end{array}$$

Since *K* is a Mercer kernel, $L_{K}$ is compact and positive. Denote $L_{K}^{r}$ as the *r*-th power of $L_{K},$ then it is well defined for any r>0 by the spectral theorem. Let ${\left\{\lambda_{i}\right\}}_{i\geq 1}$ be the eigenvalues of $L_{K},$ arranged in decreasing order. The corresponding eigenfunctions ${\left\{\phi_{i}\right\}}_{i\geq 1}$ form an orthonormal basis of $L_{\rho_{X}}^{2}$ space. Hence, the regularity space $L_{K}^{r}\left(L_{\rho_{X}}^{2}\right)$ is expressed as [18]

$$\begin{array}{c} L_{K}^{r}\left(L_{\rho_{X}}^{2}\right):=\left\{f=\sum_{i=1}^{\infty }\lambda_{j}^{r}a_{i}\phi_{i}:{∥L_{K}^{-r}f∥}_{\rho }=\sum_{i=1}^{\infty }a_{i}^{2}<\infty \right\}. \end{array}$$

It implies that for any $r_{1}>r_{2}>0$, there holds $L_{K}^{r_{1}}\left(L_{\rho_{X}}^{2}\right)\subset L_{K}^{r_{2}}\left(L_{\rho_{X}}^{2}\right).$ In particular, we know that $L_{K}^{r}\left(L_{\rho_{X}}^{2}\right)\subseteq H_{K}$ for any $r\geq \frac{1}{2}$ and $L_{K}^{\frac{1}{2}}\left(L_{\rho_{X}}^{2}\right)=H_{K}$ satisfying

(7) $$\begin{array}{c} {∥f∥}_{K}={∥L_{K}^{-\frac{1}{2}}f∥}_{\rho },\;\forall f\in H_{K}. \end{array}$$

Throughout the paper, the *regularity assumption* holds for $f_{\rho }$, i.e.,

(8) $$\begin{array}{c} f_{\rho }=L_{K}^{r}\left(g\right),\;\;for\;some\;r>0\;and\;g\in L_{\rho_{X^{2}}}^{2}, \end{array}$$

and $∥L^{-r}f_{\rho }{∥}_{\rho }={∥g∥}_{\rho }.$

This assumption is called the source condition [19] in inverse problems and it characterizes the smoothness of the target function $f_{\rho }.$ Obviously, the larger the parameter *r* is, the higher the regularity of $f_{\rho }$ is. The general source conditions considered in inverse problems usually take the form of

(9) $$\begin{array}{c} f_{\rho }=\psi \left(L_{K}\right)h,\;for\;some\;h\in H_{K} \end{array}$$

where ψ is non-decreasing and ψ(0)=0, called the index function. It is clear that when $r>\frac{1}{2},$ The above assumption is a special case of (9) with $\psi \left(L_{K}\right)=L_{K}^{r-\frac{1}{2}}$ and $h=L_{K}^{\frac{1}{2}}g.$ It should be pointed that our analysis in this work also can applied to more general cases by taking source conditions (9).

We are now in a position to state our convergence rate for (5) in $L_{\rho_{X}}^{2}$-space as well as in $H_{K}$ by choosing the step size η:=η(T). For brevity, let κ=1 without losing generality and denote the expectation $E_{z_{1},\cdots ,z_{t}}$ as $E_{t}$ for each t∈N.

**Theorem** **1.** *Define ${\left\{f_{t}\right\}}_{t=1}^{T+1}$ by (5). Suppose that the assumption (8) holds for r>0. Take $\eta =T^{-\frac{2r}{2r+1}}$ and $T>{\left(24{\left({\left(1/2e\right)}^{1/2}+1\right)}^{2}\log\left(T\right)\right)}^{\frac{2r+1}{2r}},$ then*

(10) $$\begin{array}{c} E_{T}\left[∥f_{T+1}-f_{\rho }{∥}_{\rho }^{2}\right]\leq C\max\left\{T^{-\frac{2r}{2r+1}}\log\left(T\right),T^{\frac{5}{2r+1}}\sigma^{-4}\right\} \end{array}$$

*and if $r>\frac{1}{2},$*

(11) $$\begin{array}{c} E_{T}\left[∥f_{T+1}-f_{\rho }{∥}_{K}^{2}\right]\leq C^{\prime }\max\left\{T^{-\frac{2r-1}{2r+1}},T^{\frac{5}{2r+1}}\sigma^{-4}\right\} \end{array}$$

*where the constants $C,C^{\prime }$ are independent of T,σ, and will be given in the proof.*

**Remark** **1.** *Besides the error $∥f_{T+1}-f_{\rho }{∥}_{\rho }$, the error bound (11) in $H_{K}$-norm is also given if $r>\frac{1}{2}$, i.e., $f_{\rho }\in H_{K}.$ By (3), it leads the pointwise convergence of $f_{T+1}$ to $f_{\rho }$ since for each u∈X, $|f_{T+1}\left(u\right)-f_{\rho }\left(u\right)|\leq ∥f_{T+1}-f_{\rho }{∥}_{K}.$ Compared with the global error $∥|f_{T+1}-f_{\rho }{∥}_{\rho },$ the error rate in $H_{K}$ characterizes the local performance of (5) and is much stronger. Furthermore [18], when the kernel K lies in $C^{\alpha }\left(X\times X\right)$ for some α>0, its associated RKHS $H_{K}$ can be embedded into $C^{\alpha /2}\left(X\right)$, whose partial derivative up to order α/2 are continuous with ${∥f∥}_{C^{\alpha /2}\left(X\right)}=\sum_{\left|s\right|\leq \frac{\alpha }{2}}{∥D^{\frac{\alpha }{2}}f∥}_{\infty }.$ So, the convergence in $H_{K}$ implies that $f_{T+1}$ will converge to $f_{\rho }$ in $C^{\frac{\alpha }{2}},$ that ensures the convergence of the derivatives of $f_{T+1}$ to those of $f_{\rho }.$*

**Remark** **2.** *It has been proved in [20] that the min–max optimal rate for regression problems is of order $O\left(T^{-\frac{2r}{2r+s}}\right)$ when there exists constants $C_{s}>0,$0<s≤1 such that the following effective dimension condition holds, i.e.,*

$$\begin{array}{c} Trace\left({\left(L_{K}+\lambda I\right)}^{-1}L_{K}\right)\leq C_{s}\lambda^{-s},for\;any\;\lambda >0, \end{array}$$

*where Trace(·) denotes the trace of the operator. This condition measures the complexity [15,20,21] of $H_{K}$ with respect to the marginal distribution $\rho_{X}.$ It is always satisfied with s=1 by taking the constant $C_{s}=Trace\left(L_{K}\right).$ Hence, the min–max optimal rate for capacity-independent cases is of order $O\left(T^{-\frac{2r}{2r+1}}\right)$ by taking a universal parameter s=1.*

*When $\sigma \geq T^{\frac{2r+5}{4(2r+1)}}$, we see that our convergence rate in $L_{\rho_{X}}^{2}$-norm is of order $O\left(T^{-\frac{2r}{2r+1}}\log\left(T\right)\right).$ Thus, it is nearly optimal in the capacity-independent sense that up to a logarithmic factor, it matches the min–max optimal rate above. We also find that the convergence rates (10) and (11) keep decreasing as the regularity parameter r increases. Hence, the online algorithm (5) does not suffer from the saturation phenomenon existing in Tikhonov regularization schemes [22], where the error rate of the estimators will not improve if r is out of the range (0,1]. This again shows the advantage of the online algorithm (5).*

**Remark** **3.** *Recent paper [2] investigated the approximation ability of the empirical scheme (4) over general hypothesis spaces H. This work shows that with a complexity parameter 0<β≤2, their error rate is of order $O(T^{-\frac{2}{2+\beta }})$ if the scaling parameter $\sigma =T^{\frac{1}{2+\beta }}.$ To be fair, do not take the capacity of H into consideration by taking β=2. Then, their order reduces to $O(T^{-\frac{1}{2}}),$ which is far from capacity-independent optimality and inferior to our rates.*

*In the work [17], iterative regularization techniques (alternatively called early stopping) are taken to solve the optimization problems associated with general robust losses including the correntropy induced loss $\ell_{\sigma },$ where the whole sample z are presented at each iteration. In their analysis, under the polynomial decay of the eigenvalues $\{\lambda_{i}\},$ that is, there exists some constants $C_{b}>0$ and b≥1 such that*

$$\begin{array}{c} \lambda_{i}\leq c_{b}i^{-b},\;\forall i\geq 1, \end{array}$$

*the obtained rate is $O(T^{-\frac{2br}{2br+1}})$ if $r\geq \frac{1}{2}$, else, it is $O(T^{-\frac{2br}{b+1}}).$ This decay is also a measurement for the complexity of $H_{K},$ please refer to [21]. Recall that the compactness of X implies that $\sum_{i}\lambda_{i}<\infty$ and $\lambda_{i}\leq ci^{-1}$ for some c>0. So, their rate for capacity-independent cases is $O(T^{-\frac{2r}{2r+1}})$ if $r\geq \frac{1}{2}$, else, it is $O(T^{-r}).$ We can see that our results in (10) are superior in the case $0<r<\frac{1}{2}.$ It shows in theory that the online algorithm (5) for MCC can achieve better approximation rate when $f_{\rho }$ is not in $H_{K}.$*

**Remark** **4.** *It is easy to check that the roots of the second derivative of $\ell_{\sigma }$ is ±σ, i.e., when |f(x)−y|<σ, this loss is convex and behaves as the least squares loss; when |f(x)−y|≥σ, the loss function becomes concave and rapidly tends to be flat as the value of |f(x)−y| goes to infinity. It implies that $\ell_{\sigma }$ satisfies the redescending property, and with a suitable chosen scaling parameter σ, $\ell_{\sigma }$ can reject gross outliers while keeping a prediction accuracy. In Theorem 1, we observe that σ should be large enough to guarantee the nice convergence, which coincides with the work in [2]. They also pointed that too small σ may prevent the estimator to converge to $f_{\rho }.$ In a recent paper [23], correntropy with small σ is interpreted as modal regression. According to the above discussions and empirical studies [2,14,17], we conclude that the value of σ would determine the learning target and a moderate σ may be more appropriate for balancing the convergence and robustness in practice.*

Based on the above remarks, we see that the convergence rate of online kernel-based MCC is comparable to that of the least squares that has appeared in the literature [24]. Meanwhile, MCC’s redescending property will produce robustness to various outliers including sub-Gaussain, Student’s *t*-distribution, and Cauchy distribution. These all shows the superiority of MCC in a variety of applications, such as clustering, classification and feature selection [14]. At the end of this section, we would like to point out that although our work is carried out under the boundness condition of Y, it can be extended to more general situations such as the moment conditions [20].

## 3. Proofs of Main Result

In this section, we prove our main results in Theorem 1. First, we derive the uniform bound for the iteration sequence ${\left\{f_{t}\right\}}_{t=1}^{T+1}$ by (5).

**Lemma** **1.** *Define ${\left\{f_{t}\right\}}_{t=1}^{T+1}$ by (5). If 0<η≤1, then*

(12) $$\begin{array}{c} ∥f_{t}{∥}_{K}\leq M\eta^{\frac{1}{2}}{\left(t-1\right)}^{\frac{1}{2}},\;t\in N. \end{array}$$

**Proof.**  We prove (12) by induction. It is trivial that (12) holds for t=1. Suppose (12) holds for t≥2. Notice that $\ell_{\sigma }^{\prime }\left(f_{t}\left(x_{t}\right),y_{t}\right)=G\left(-\frac{{\left(f_{t}\left(x_{t}\right)-y_{t}\right)}^{2}}{2\sigma^{2}}\right)\left[f_{t}\left(x_{t}\right)-y_{t}\right].$ Write (12) as $f_{t+1}=f_{t}-\eta H_{t}$ where $H_{t}=G\left(-\frac{{\left(f_{t}\left(x_{t}\right)-y_{t}\right)}^{2}}{2\sigma^{2}}\right)\left[f_{t}\left(x_{t}\right)-y_{t}\right]K_{x_{t}}.$ Then by (2),

$$\begin{array}{c} ∥f_{t+1}{∥}_{K}^{2}=∥f_{t}{∥}_{K}^{2}-2\eta {\langle f_{t},H_{t}\rangle }_{K}+\eta^{2}{∥H_{t}∥}_{K}^{2}\\ =∥f_{t}{∥}_{K}^{2}-2\eta G\left(-\frac{{\left(f_{t}\left(x_{t}\right)-y_{t}\right)}^{2}}{2\sigma^{2}}\right)\left[f_{t}\left(x_{t}\right)-y_{t}\right]f_{t}\left(x_{t}\right)+\eta^{2}{∥H_{t}∥}_{K}^{2} \end{array}$$

and

$$\begin{array}{cc} ∥H_{t}{∥}_{K}^{2} & =G\left(-\frac{{\left(f_{t}\left(x_{t}\right)-y_{t}\right)}^{2}}{\sigma^{2}}\right){\left[f_{t}\left(x_{t}\right)-y_{t}\right]}^{2}K_{\left(x_{t},x_{t}\right)}\\ & \leq G\left(-\frac{{\left(f_{t}\left(x_{t}\right)-y_{t}\right)}^{2}}{\sigma^{2}}\right){\left[f_{t}\left(x_{t}\right)-y_{t}\right]}^{2}. \end{array}$$

Then, we have

$$\begin{array}{c} ∥f_{t+1}{∥}_{K}^{2}\leq {∥f_{t}∥}_{K}^{2}+\eta \left\{\eta G\left(-\frac{{\left(f_{t}\left(x_{t}\right)-y_{t}\right)}^{2}}{2\sigma^{2}}\right){\left[f_{t}\left(x_{t}\right)-y_{t}\right]}^{2}-2\left(f_{t}\left(x_{t}\right)-y_{t}\right)f_{t}\left(x_{t}\right)\right\}G\left(-\frac{{\left(f_{t}\left(x_{t}\right)-y_{t}\right)}^{2}}{2\sigma^{2}}\right). \end{array}$$

For the part of the above inequality, we have

$$\begin{array}{c} \eta G\left(-\frac{{\left(f_{t}\left(x_{t}\right)-y_{t}\right)}^{2}}{2\sigma^{2}}\right){\left[f_{t}\left(x_{t}\right)-y_{t}\right]}^{2}-2\left(f_{t}\left(x_{t}\right)-y_{t}\right)f_{t}\left(x_{t}\right)\\ =\left(\eta G\left(-\frac{{\left(f_{t}\left(x_{t}\right)-y_{t}\right)}^{2}}{2\sigma^{2}}\right)-2\right){\left(\left(f_{t}\left(x_{t}\right)-y_{t}\right)-\frac{y_{t}}{\eta G\left(-\frac{{\left(f_{t}\left(x_{t}\right)-y_{t}\right)}^{2}}{2\sigma^{2}}\right)-2}\right)}^{2}\\ +\frac{y_{t}^{2}}{2-\eta G\left(-\frac{{\left(f_{t}\left(x_{t}\right)-y_{t}\right)}^{2}}{2\sigma^{2}}\right)}. \end{array}$$

Since η≤1, it follows that $\eta G\left(-\frac{{\left(f_{t}\left(x_{t}\right)-y_{t}\right)}^{2}}{2\sigma^{2}}\right)-2<0$ and $2-\eta G\left(-\frac{{\left(f_{t}\left(x_{t}\right)-y_{t}\right)}^{2}}{2\sigma^{2}}\right)\geq 1.$ Recall that |y|≤M for all y∈Y, then

$$\begin{array}{c} \eta G\left(-\frac{{\left(f_{t}\left(x_{t}\right)-y_{t}\right)}^{2}}{2\sigma^{2}}\right){\left[f_{t}\left(x_{t}\right)-y_{t}\right]}^{2}-2\left(f_{t}\left(x_{t}\right)-y_{t}\right)f_{t}\left(x_{t}\right)\leq \frac{y_{t}^{2}}{2-\eta G\left(-\frac{{\left(f_{t}\left(x_{t}\right)-y_{t}\right)}^{2}}{2\sigma^{2}}\right)}\leq M^{2}. \end{array}$$

Based on the above analysis,

$$\begin{array}{c} ∥f_{t+1}{∥}_{K}^{2}\leq ∥f_{t}{∥}_{K}^{2}+\eta M^{2}G\left(-\frac{{\left(f_{t}\left(x_{t}\right)-y_{t}\right)}^{2}}{2\sigma^{2}}\right)\leq {∥f_{t}∥}_{K}^{2}+\eta M^{2}\leq M^{2}\eta \left(t-1\right)+\eta M^{2}=M^{2}\eta t. \end{array}$$

Then the proof is completed. □

Next, we will establish a proposition which is crucial to prove the convergence rates in Theorem 1. It is closely related to the generalization error of $f_{t}.$ Define the *generalization error*E(f) for any measurable function f:X→R by

$$\begin{array}{c} E\left(f\right)=\int_{Z}{\left(f\left(x\right)-y\right)}^{2}d\rho . \end{array}$$

The regression function $f_{\rho }$ that we want to learn or approximate is a minimizer of E(f) , that is

$$\begin{array}{c} f_{\rho }=\arg\min\left\{E\left(f\right):f\;is\;a\;measurable\;function\;from\;X\;to\;Y\right\}. \end{array}$$

A simple computation yields the relation for f:X→R

(13) $$\begin{array}{c} ∥f-f_{\rho }{∥}_{\rho }^{2}=E\left(f\right)-E\left(f_{\rho }\right). \end{array}$$

For brevity, set the operator $\pi_{k}^{t}\left(L_{K}\right):=\prod_{j=k}^{t}\left(I-\eta L_{K}\right)$ for k,t∈N and $\pi_{t+1}^{t}\left(L_{K}\right):=I.$

**Proposition** **1.** *Define ${\left\{f_{t}\right\}}_{t=1}^{T+1}$ by (5). If the step size 0<η<1, then we have*

(14) $$\begin{array}{cc} E_{T}\left[∥f_{T+1}-f_{\rho }{∥}_{\rho }^{2}\right] & \leq 2{∥\pi_{1}^{T}\left(L_{K}\right)f_{\rho }∥}_{\rho }^{2}+2\eta^{2}\sum_{t=1}^{T}\frac{2{\left({\left(1/2e\right)}^{1/2}+1\right)}^{2}}{1+\eta (T-t)}E_{t-1}\left[E(f_{t})\right]\\ & +2E_{T}\left[{∥\eta \sum_{t=1}^{T}\pi_{t+1}^{T}\left(L_{K}\right)\Delta_{t}∥}_{\rho }^{2}\right], \end{array}$$

*furthermore, if $f_{\rho }\in H_{K},$*

(15) $$\begin{array}{cc} E_{T}\left[∥f_{T+1}-f_{\rho }{∥}_{K}^{2}\right] & \leq 2{∥\pi_{1}^{T}\left(L_{K}\right)f_{\rho }∥}_{K}^{2}+2\eta^{2}\sum_{t=1}^{T}E_{t-1}\left[E(f_{t})\right]+2E_{T}\left[{∥\eta \sum_{t=1}^{T}\pi_{t+1}^{T}\left(L_{K}\right)\Delta_{t}∥}_{K}^{2}\right], \end{array}$$

*where $\Delta_{t}$ is defined in the proof.*

**Proof.**  Denote

(16) $$\begin{array}{cc} \Delta_{t} & =\left[G\left(0\right)-G\left(-\frac{{\left(f_{t}\left(x_{t}\right)-y_{t}\right)}^{2}}{2\sigma^{2}}\right)\right]\left[f_{t}\left(x_{t}\right)-y_{t}\right]K_{x_{t}}\\ & =\left[1-G\left(-\frac{{\left(f_{t}\left(x_{t}\right)-y_{t}\right)}^{2}}{2\sigma^{2}}\right)\right]\left[f_{t}\left(x_{t}\right)-y_{t}\right]K_{x_{t}} \end{array}$$

and define a random variable $\xi \left(f_{t},z_{t}\right):=L_{K}\left(f_{t}-f_{\rho }\right)-\left(f_{t}\left(x_{t}\right)-y_{t}\right)K_{x_{t}.}$

By (5), we have that for any t∈N,

$$\begin{array}{c} f_{t+1}-f_{\rho }=f_{t}-f_{\rho }-\eta \left[f_{t}\left(x_{t}\right)-y_{t}\right]K_{x_{t}}+\eta \Delta_{t}=\left(I-\eta L_{K}\right)\left(f_{t}-f_{\rho }\right)+\eta \xi \left(f_{t},z_{t}\right)+\eta \Delta_{t}. \end{array}$$

Applying the above equality iteratively from t=T to t=1, we get that by $f_{1}=0,$

(17) $$\begin{array}{c} f_{T+1}-f_{\rho }=-\pi_{1}^{T}\left(L_{K}\right)f_{\rho }+\eta \sum_{t=1}^{T}\pi_{t+1}^{T}\left(L_{K}\right)\xi \left(f_{t},z_{t}\right)+\eta \sum_{t=1}^{T}\pi_{t+1}^{T}\left(L_{K}\right)\Delta_{t}. \end{array}$$

It follows from the elementary inequality that $∥g_{1}+g_{2}{∥}_{\rho }^{2}\leq 2∥g_{1}{∥}_{\rho }^{2}+2{∥g_{2}∥}_{\rho }^{2}$ for any $g_{1},g_{2}\in L_{\rho_{X}}^{2},$ that

(18) $$\begin{array}{c} E_{T}\left[∥f_{T+1}-f_{\rho }{∥}_{\rho }^{2}\right]\leq 2E_{T}\left[{∥-\pi_{1}^{T}\left(L_{K}\right)f_{\rho }+\eta \sum_{t=1}^{T}\pi_{t+1}^{T}\left(L_{K}\right)\xi \left(f_{t},z_{t}\right)∥}_{\rho }^{2}\right]+2E_{T}\left[{∥\eta \sum_{t=1}^{T}\pi_{t+1}^{T}\left(L_{K}\right)\Delta_{t}∥}_{\rho }^{2}\right]. \end{array}$$

To prove (14), we consider the part of the first term on the right-hand side of (18)

(19) $$\begin{array}{c} E_{T}\left[{∥-\pi_{1}^{T}\left(L_{K}\right)f_{\rho }+\eta \sum_{t=1}^{T}\pi_{t+1}^{T}\left(L_{K}\right)\xi \left(f_{t},z_{t}\right)∥}_{\rho }^{2}\right]={∥\pi_{1}^{T}\left(L_{K}\right)f_{\rho }∥}_{\rho }^{2}\\ +E_{T}\left[{∥\eta \sum_{t=1}^{T}\pi_{t+1}^{T}\left(L_{K}\right)\xi \left(f_{t},z_{t}\right)∥}_{\rho }^{2}\right]-2E_{T}\left[{\langle \pi_{1}^{T}\left(L_{K}\right)f_{\rho },\eta \sum_{t=1}^{T}\pi_{t+1}^{T}\left(L_{K}\right)\xi \left(f_{t},z_{t}\right)\rangle }_{\rho }\right]. \end{array}$$

Observe that $f_{t}$ is only dependent on $\left\{z_{1},\cdots ,z_{t-1}\right\}$, not on $z_{t}.$ Thus, by the fact that $\int_{X}yd\rho =f_{\rho }\left(x\right),$ we have

(20) $$\begin{array}{c} E_{z_{t}}\left[\xi (f_{t},z_{t})\right]=0,\;t=1,\cdots ,T. \end{array}$$

We consider the second term on the right-hand side of (19). It can be rewritten as

$$\begin{array}{c} E_{T}\left[{∥\eta \sum_{t=1}^{T}\pi_{t+1}^{T}\left(L_{K}\right)\xi \left(f_{t},z_{t}\right)∥}_{\rho }^{2}\right]=\eta^{2}\sum_{t=1}^{T}\sum_{l=1}^{T}E_{T}{\langle \pi_{t+1}^{T}\left(L_{K}\right)\xi \left(f_{t},z_{t}\right),\pi_{l+1}^{T}\left(L_{K}\right)\xi \left(f_{l},z_{l}\right)\rangle }_{\rho }. \end{array}$$

When t<l≤T, by (20),

$$\begin{array}{c} E_{T}{\langle \pi_{t+1}^{T}\left(L_{K}\right)\xi \left(f_{t},z_{t}\right),\pi_{l+1}^{T}\left(L_{K}\right)\xi \left(f_{l},z_{l}\right)\rangle }_{\rho }=E_{l-1}E_{z_{l}}{\langle \pi_{t+1}^{T}\left(L_{K}\right)\xi \left(f_{t},z_{t}\right),\pi_{l+1}^{T}\left(L_{K}\right)\xi \left(f_{l},z_{l}\right)\rangle }_{\rho }\\ =E_{l-1}{\langle \pi_{l+1}^{T}\left(L_{K}\right)\pi_{t+1}^{T}\left(L_{K}\right)\xi \left(f_{t},z_{t}\right),E_{z_{l}}\xi \left(f_{l},z_{l}\right)\rangle }_{\rho }=0. \end{array}$$

Obviously, the above equality holds for l<t≤T. So, with (7), we get

$$\begin{array}{c} \eta^{2}\sum_{t=1}^{T}\sum_{l=1}^{T}E_{T}{\langle \pi_{t+1}^{T}\left(L_{K}\right)\xi \left(f_{t},z_{t}\right),\pi_{l+1}^{T}\left(L_{K}\right)\xi \left(f_{l},z_{l}\right)\rangle }_{\rho }\\ =\eta^{2}\sum_{t=1}^{T}E_{t}\left[{∥\pi_{t+1}^{T}\left(L_{K}\right)\xi \left(f_{t},z_{t}\right)∥}_{\rho }^{2}\right]\leq \eta^{2}\sum_{t=1}^{T}{∥\pi_{t+1}^{T}\left(L_{K}\right)L_{K}^{\frac{1}{2}}∥}^{2}E_{t}\left[{∥L_{K}^{-\frac{1}{2}}\xi \left(f_{t},z_{t}\right)∥}_{\rho }^{2}\right]\\ =\eta^{2}\sum_{t=1}^{T}{∥\pi_{t+1}^{T}\left(L_{K}\right)L_{K}^{\frac{1}{2}}∥}^{2}E_{t}\left[{∥\xi (f_{t},z_{t})∥}_{K}^{2}\right]. \end{array}$$

To bound $E_{t}\left[{∥\xi (f_{t},z_{t})∥}_{K}^{2}\right],$ we have

$$\begin{array}{c} E_{t}\left[{∥\xi (f_{t},z_{t})∥}_{K}^{2}\right]=E_{t}\left[{∥\left(f_{t}\left(x_{t}\right)-y_{t}\right)K_{x_{t}}∥}_{K}^{2}\right]-{∥E_{t}\left[\left(f_{t}\left(x_{t}\right)-y_{t}\right)K_{x_{t}}\right]∥}_{K}^{2}\\ \leq E_{t-1}E_{z_{t}}\left[{∥\left(f_{t}\left(x_{t}\right)-y_{t}\right)K_{x_{t}}∥}_{K}^{2}\right]\leq E_{t-1}E_{z_{t}}\left[{\left(f_{t}\left(x_{t}\right)-y_{t}\right)}^{2}\right]=E_{t-1}\left[E(f_{t})\right] \end{array}$$

where the last inequality is derived from (3). Applying Lemma A1 with $\beta =\frac{1}{2}$, l=t+1 and k=T, we have

$$\begin{array}{c} \sum_{t=1}^{T}{∥\pi_{t+1}^{T}\left(L_{K}\right)L_{K}^{\frac{1}{2}}∥}^{2}=\sum_{t=1}^{T-1}{∥\pi_{t+1}^{T}\left(L_{K}\right)L_{K}^{\frac{1}{2}}∥}^{2}+{∥\pi_{T+1}^{T}\left(L_{K}\right)L_{K}^{\frac{1}{2}}∥}^{2}\\ \leq \sum_{t=1}^{T-1}\frac{2{\left({\left(1/2e\right)}^{1/2}+1\right)}^{2}}{1+\eta (T-t)}+1\leq \sum_{t=1}^{T}\frac{2{\left({\left(1/2e\right)}^{1/2}+1\right)}^{2}}{1+\eta (T-t)}. \end{array}$$

Based on the above analysis, we have

(21) $$\begin{array}{c} E_{T}\left[{∥\eta \sum_{t=1}^{T}\pi_{t+1}^{T}\left(L_{K}\right)\xi \left(f_{t},z_{t}\right)∥}_{\rho }^{2}\right]\leq \eta^{2}\sum_{t=1}^{T}\frac{2{\left({\left(1/2e\right)}^{1/2}+1\right)}^{2}}{1+\eta (T-t)}E_{t-1}\left[E(f_{t})\right]. \end{array}$$

Now, we estimate the last term on the right-hand side of (19). Using (20) again, we have

(22) $$\begin{array}{c} E_{T}\left[{\langle \pi_{1}^{T}\left(L_{K}\right)f_{\rho },\eta \sum_{t=1}^{T}\pi_{t+1}^{T}\left(L_{K}\right)\xi \left(f_{t},z_{t}\right)\rangle }_{\rho }\right]\\ ={\langle \pi_{1}^{T}\left(L_{K}\right)f_{\rho },\eta \sum_{t=1}^{T}\pi_{t+1}^{T}\left(L_{K}\right)E_{t-1}E_{z_{t}}\left[\xi (f_{t},z_{t})\right]\rangle }_{\rho }=0. \end{array}$$

Plugging (21) and (22) into (19), we get

(23) $$\begin{array}{c} E_{T}\left[{∥-\pi_{1}^{T}\left(L_{K}\right)f_{\rho }+\eta \sum_{t=1}^{T}\pi_{t+1}^{T}\left(L_{K}\right)\xi \left(f_{t},z_{t}\right)∥}_{\rho }^{2}\right]={∥\pi_{1}^{T}\left(L_{K}\right)f_{\rho }∥}_{\rho }^{2}\\ +\eta^{2}\sum_{t=1}^{T}\frac{2{\left({\left(1/2e\right)}^{1/2}+1\right)}^{2}}{1+\eta (T-t)}E_{t-1}\left[E(f_{t})\right]. \end{array}$$

This together with (18) yields the desired conclusion (14).

Now we turn to bound $f_{T+1}-f_{\rho }$ in $H_{K}$-norm. By (17) again, we have

$$\begin{array}{c} E_{T}\left[∥f_{T+1}-f_{\rho }{∥}_{K}^{2}\right]\leq 2E_{T}\left[{∥-\pi_{1}^{T}\left(L_{K}\right)f_{\rho }+\eta \sum_{t=1}^{T}\pi_{t+1}^{T}\left(L_{K}\right)\xi \left(f_{t},z_{t}\right)∥}_{K}^{2}\right]+2E_{T}\left[{∥\eta \sum_{t=1}^{T}\pi_{t+1}^{T}\left(L_{K}\right)\Delta_{t}∥}_{K}^{2}\right]. \end{array}$$

Following the similar procedure in estimating (14), we also get

$$\begin{array}{c} E_{T}\left[∥f_{T+1}-f_{\rho }{∥}_{K}^{2}\right]\leq 2{∥\pi_{1}^{T}\left(L_{K}\right)f_{\rho }∥}_{K}^{2}+2\eta^{2}\sum_{t=1}^{T}{∥\pi_{t+1}^{T}\left(L_{K}\right)∥}^{2}E_{t-1}\left[E(f_{t})\right]+2E_{T}\left[{∥\eta \sum_{t=1}^{T}\pi_{t+1}^{T}\left(L_{K}\right)\Delta_{t}∥}_{K}^{2}\right]. \end{array}$$

Noticing that $∥\pi_{t+1}^{T}\left(L_{K}\right)∥\leq 1,$ then the bound (15) is obtained. □

Based on the error bounds of $f_{T+1}-f_{\rho }$ in Proposition 1, we need to estimate the generalization error $E(f_{t}).$

**Lemma** **2.** *Define ${\left\{f_{t}\right\}}_{t=1}^{T+1}$ by (5). If*

(24) $$\begin{array}{c} 0<\eta \leq \min\left\{1,\frac{1}{8}{\left({\left(1/2e\right)}^{1/2}+1\right)}^{-2}{\left(\log\left(et\right)+1\right)}^{-1}\right\}, \end{array}$$

*then for t≥2,*

(25) $$\begin{array}{c} E_{t-1}\left[E(f_{t})\right]\leq 2E\left(f_{\rho }\right)+4{∥f_{\rho }∥}_{\rho }^{2}+64\eta^{2}\sigma^{-4}{\left(t-1\right)}^{2}{\left(\underset{1\leq k\leq t-1}{\sup}\{∥f_{k}{∥}_{K},M\}\right)}^{6}. \end{array}$$

**Proof.**  We shall prove (25) by induction. Obviously, (25) holds for t=2. Suppose (25) holds for t≥2. Applying (14) with T=t, then

(26) $$\begin{array}{cc} E_{t}\left[∥f_{t+1}-f_{\rho }{∥}_{\rho }^{2}\right] & \leq 2{∥\pi_{1}^{t}\left(L_{K}\right)f_{\rho }∥}_{\rho }^{2}+2\eta^{2}\sum_{k=1}^{t}\frac{2{\left({\left(1/2e\right)}^{1/2}+1\right)}^{2}}{1+\eta (t-k)}E_{k-1}\left[E(f_{k})\right]+2E_{t}\left[{∥\eta \sum_{k=1}^{t}\pi_{k+1}^{t}\left(L_{K}\right)\Delta_{k}∥}_{\rho }^{2}\right]\\ & \leq 2{∥\pi_{1}^{t}\left(L_{K}\right)f_{\rho }∥}_{\rho }^{2}+2\eta^{2}\sum_{k=1}^{t}\frac{2{\left({\left(1/2e\right)}^{1/2}+1\right)}^{2}}{1+\eta (t-k)}E_{k-1}\left[E(f_{k})\right]+2\eta^{2}{\left(\sum_{k=1}^{t}∥\pi_{k+1}^{t}\left(L_{K}\right)∥∥\Delta_{k}{∥}_{\infty }\right)}^{2}. \end{array}$$

Since the Gaussian *G* is Lipschitz continuous, we have that for each 1≤k≤T,

$$\begin{array}{cc} ∥\Delta_{k}{∥}_{\infty } & \leq {∥\left[G\left(0\right)-G\left(-\frac{{\left(f_{k}\left(x_{k}\right)-y_{t}\right)}^{2}}{2\sigma^{2}}\right)\right]\left[f_{k}\left(x_{k}\right)-y_{k}\right]K_{x_{k}}∥}_{\infty }\\ & \leq \left|\left[G\left(0\right)-G\left(-\frac{{\left(f_{k}\left(x_{k}\right)-y_{k}\right)}^{2}}{2\sigma^{2}}\right)\right]\left[f_{t}\left(x_{k}\right)-y_{k}\right]\right|{∥K_{x_{k}}∥}_{K}\\ & \leq \frac{{\left(f_{k}\left(x_{k}\right)-y_{k}\right)}^{2}}{2\sigma^{2}}\left|f_{k}\left(x_{k}\right)-y_{k}\right|\leq \frac{(∥f_{k}{{∥}_{\infty }+M)}^{3}}{2\sigma^{2}}\leq \frac{(∥f_{k}{{∥}_{K}+M)}^{3}}{2\sigma^{2}} \end{array}$$

where the last inequality is derived from (3).

Notice that by 0<η≤1, there holds $∥\pi_{k}^{t}\left(L_{K}\right)∥\leq \prod_{l=k}^{t}∥I-\eta L_{K}∥\leq 1$ for each 1≤k≤t≤T. Then the last term on the right-hand side of (26) is bounded as

(27) $$\begin{array}{c} 2\eta^{2}{\left(\sum_{k=1}^{t}∥\pi_{k+1}^{t}\left(L_{K}\right)∥∥\Delta_{k}{∥}_{\infty }\right)}^{2}\leq 32\eta^{2}\sigma^{-4}t^{2}{\left(\underset{1\leq k\leq t}{\sup}\{∥f_{k}{∥}_{K},M\}\right)}^{6}. \end{array}$$

For the first term $2{∥\pi_{1}^{t}\left(L_{K}\right)f_{\rho }∥}_{\rho }^{2}$, it is easy to get that $2{∥\pi_{1}^{t}\left(L_{K}\right)f_{\rho }∥}_{\rho }^{2}\leq 2{∥f_{\rho }∥}_{\rho }^{2}.$

Putting the above estimates into (26) and using the relation (13) with $f=f_{t+1},$ we have

(28) $$\begin{array}{c} E_{t}\left[E(f_{t+1})\right]=E_{t}\left[∥f_{t+1}-f_{\rho }{∥}_{\rho }^{2}\right]+E\left(f_{\rho }\right)\\ \leq E\left(f_{\rho }\right)+2{∥f_{\rho }∥}_{\rho }^{2}+32\eta^{2}\sigma^{-4}t^{2}{\left(\underset{1\leq k\leq t}{\sup}\{∥f_{k}{∥}_{K},M\}\right)}^{6}+2\eta^{2}\sum_{k=1}^{t}\frac{2{\left({\left(1/2e\right)}^{1/2}+1\right)}^{2}}{1+\eta (t-k)}E_{k-1}\left[E(f_{k})\right]\\ \leq E\left(f_{\rho }\right)+2{∥f_{\rho }∥}_{\rho }^{2}+32\eta^{2}\sigma^{-4}t^{2}{\left(\underset{1\leq k\leq t}{\sup}\{∥f_{k}{∥}_{K},M\}\right)}^{6}\\ +2\eta^{2}\sum_{k=1}^{t}\frac{2{\left({\left(1/2e\right)}^{1/2}+1\right)}^{2}}{1+\eta (t-k)}\left(2E\left(f_{\rho }\right)+4{∥f_{\rho }∥}_{\rho }^{2}+64\eta^{2}\sigma^{-4}{\left(t-1\right)}^{2}{\left(\underset{1\leq k\leq t-1}{\sup}\{∥f_{k}{∥}_{K},M\}\right)}^{6}\right). \end{array}$$

By the restriction (24) of η and Lemma A3, we know that

$$\begin{array}{c} 2\eta^{2}\sum_{k=1}^{t}\frac{2{\left({\left(1/2e\right)}^{1/2}+1\right)}^{2}}{1+\eta (t-k)}\leq 4\eta {\left({\left(1/2e\right)}^{1/2}+1\right)}^{2}\left(\log\left(et\right)+1\right)\leq \frac{1}{2}. \end{array}$$

Plugging it into (28), we have

$$\begin{array}{c} E_{t}\left[E(f_{t+1})\right]\leq E\left(f_{\rho }\right)+2{∥f_{\rho }∥}_{\rho }^{2}+32\eta^{2}\sigma^{-4}t^{2}{\left(\underset{1\leq k\leq t}{\sup}\{∥f_{k}{∥}_{K},M\}\right)}^{6}\\ +\frac{1}{2}\left(2E\left(f_{\rho }\right)+4{∥f_{\rho }∥}_{\rho }^{2}+64\eta^{2}\sigma^{-4}{\left(t-1\right)}^{2}{\left(\underset{1\leq k\leq t-1}{\sup}\{∥f_{k}{∥}_{K},M\}\right)}^{6}\right)\\ \leq 2E\left(f_{\rho }\right)+4{∥f_{\rho }∥}_{\rho }^{2}+64\eta^{2}\sigma^{-4}t^{2}{\left(\underset{1\leq k\leq t}{\sup}\{∥f_{k}{∥}_{K},M\}\right)}^{6}. \end{array}$$

Then the proof is completed. □

With these preliminaries in place, we shall prove our main results.

**Proof** **of Theorem 1.** We shall prove Theorem 1 by Proposition 1. First, we will use (14) to estimate the error rate for (5) in $L_{\rho_{X}}^{2}$-space. For the first term on the right-hand side of (14), applying Lemma A2 with $f=f_{\rho }$ and $\eta =T^{-\frac{2r}{2r+1}}$, we have that

$$\begin{array}{c} {∥\pi_{1}^{T}\left(L_{K}\right)f_{\rho }∥}_{\rho }^{2}\leq 4{\left({\left(r/e\right)}^{r}+1\right)}^{2}∥L_{K}^{-r}f_{\rho }{∥}_{\rho }^{2}T^{-\frac{2r}{2r+1}}=4{\left({\left(r/e\right)}^{r}+1\right)}^{2}{∥g∥}_{\rho }^{2}T^{-\frac{2r}{2r+1}}. \end{array}$$

For the second term on the right-hand side of (14), the choice of η and *T* in Theorem 1 implies that the restriction (24) holds. Then we can put the bound (12) into (25) and get that for t≥2

$$\begin{array}{c} E_{t-1}\left[E(f_{t})\right]\leq 2E\left(f_{\rho }\right)+4{∥f_{\rho }∥}_{\rho }^{2}+64M^{6}\sigma^{-4}\eta^{5}{\left(t-1\right)}^{5}\\ \leq \left(2E\left(f_{\rho }\right)+4{∥f_{\rho }∥}_{\rho }^{2}+64M^{6}\right)\left(1+\sigma^{-4}\eta^{5}{\left(t-1\right)}^{5}\right)\\ \leq \left(2E\left(f_{\rho }\right)+4{∥f_{\rho }∥}_{\rho }^{2}+64M^{6}\right)\left(1+\sigma^{-4}\eta^{5}T^{5}\right):=c_{M,\rho }\left(1+\sigma^{-4}T^{\frac{5}{2r+1}}\right). \end{array}$$

This together with Lemma A3 yields that

$$\begin{array}{cc} \eta^{2}\sum_{t=1}^{T}\frac{2{\left({\left(1/2e\right)}^{1/2}+1\right)}^{2}}{1+\eta (T-t)}E_{t-1}\left[E(f_{t})\right] & \leq 2{\left({\left(1/2e\right)}^{1/2}+1\right)}^{2}c_{M,\rho }\eta \left(\log\left(eT\right)+1\right)\left(1+\sigma^{-4}T^{\frac{5}{2r+1}}\right)\\ & \leq 4{\left({\left(1/2e\right)}^{1/2}+1\right)}^{2}c_{M,\rho }\log\left(T\right)\left(T^{-\frac{2r}{2r+1}}+\sigma^{-4}T^{\frac{5-2r}{2r+1}}\right). \end{array}$$

Finally, we bound the last term on the right-hand side of (14). Notice that

$$\begin{array}{c} {∥\eta \sum_{t=1}^{T}\pi_{t+1}^{T}\left(L_{K}\right)\Delta_{t}∥}_{\rho }\leq \eta \sum_{t=1}^{T}∥\pi_{t+1}^{t}\left(L_{K}\right)∥∥\Delta_{t}{∥}_{\infty }. \end{array}$$

Then, using the estimate (27) and the bound (12) of $\left\{f_{t}\right\}$, we have

$$\begin{array}{c} E_{T}\left[{∥\eta \sum_{t=1}^{T}\pi_{t+1}^{T}\left(L_{K}\right)\Delta_{t}∥}_{\rho }^{2}\right]\leq \eta^{2}{\left(\sum_{t=1}^{T}∥\pi_{t+1}^{T}\left(L_{K}\right)∥∥\Delta_{t}{∥}_{\infty }\right)}^{2}\\ \leq 16\eta^{2}\sigma^{-4}t^{2}{\left(\underset{1\leq t\leq T}{\sup}\{∥f_{t}{∥}_{K},M\}\right)}^{6}\leq 16M^{6}\sigma^{-4}T^{\frac{5}{2r+1}}. \end{array}$$

Based on the above analysis, the conclusion (10) is obtained by taking

$$C=8{\left({\left(r/e\right)}^{r}+1\right)}^{2}{∥g∥}_{\rho }^{2}+16{\left({\left(1/2e\right)}^{1/2}+1\right)}^{2}c_{M,\rho }+32M^{6}.$$

Similarity, we can get the conclusion (11) by taking

$$C^{\prime }=8{\left({\left(\left(2r-1\right)/2e\right)}^{r-\frac{1}{2}}+1\right)}^{2}{∥g∥}_{\rho }^{2}+8c_{M,\rho }+32M^{6}.$$

 □

## Appendix A. Useful Lemmas

The following two lemmas are slightly modified forms of Lemma 3, Lemma 7 in [24], respectively.

**Lemma** **A1.**
*Let β>0 and 0<η≤1. Then for any t∈[l,k], there holds*

$$\begin{array}{c} ∥\pi_{l}^{k}\left(L_{K}\right)L_{K}^{\beta }{∥}^{2}\leq \frac{2{\left({\left(\beta /e\right)}^{\beta }+1\right)}^{2}}{1+\eta^{2\beta }{\left(k-l+1\right)}^{2\beta }}. \end{array}$$

**Lemma** **A2.**
*If $f\in L_{K}^{r}\left(L_{\rho_{X}}^{2}\right)$ for some r>0, then*

$$\begin{array}{c} ∥\pi_{1}^{T}\left(L_{K}\right){f∥}_{\rho }\leq 2\left({\left(r/e\right)}^{r}+1\right){∥L_{K}^{-r}f∥}_{\rho }\eta^{-r}T^{-r}. \end{array}$$

*In addition, if $r>\frac{1}{2},$ then*

$$\begin{array}{c} ∥\pi_{1}^{T}\left(L_{K}\right){f∥}_{K}\leq 2\left({\left(\left(2r-1\right)/2e\right)}^{r-\frac{1}{2}}+1\right){∥L_{K}^{-r}f∥}_{\rho }\eta^{-r+\frac{1}{2}}T^{-r+\frac{1}{2}}. \end{array}$$

**Lemma** **A3.**
*For any 0<η≤1, there holds for t≥2,*

$$\begin{array}{c} \sum_{k=1}^{t}\frac{1}{1+\eta (t-k)}\leq \eta^{-1}\left(\log\left(et\right)+1\right). \end{array}$$

**Proof.** By the elementary inequality $\sum_{k=1}^{t}k^{-1}\leq \log e\left(t+1\right),$ we know that for t≥2,

$$\begin{array}{cc} \sum_{k=1}^{t}\frac{1}{1+\eta (t-k)} & =\sum_{k=1}^{t-1}\frac{1}{1+\eta (t-k)}+1\leq \eta^{-1}\sum_{k=1}^{t-1}{\left(t-k\right)}^{-1}+1=\eta^{-1}\sum_{k=1}^{t-1}\frac{1}{k}+1\\ & \leq \eta^{-1}\log\left(et\right)+1\leq \eta^{-1}\left(\log\left(et\right)+1\right). \end{array}$$

Then the proof is completed. □

## References

[1] Santamaria I., Pokharel P.P., Principe J.C. (2006). Generalized correlation function: Definition, properties, and application to blind equalization. IEEE Trans. Signal Process..

[2] Feng Y.L., Huang X.L., Shi L., Yang Y.N., Suykens J.A.K. (2015). Learning with the Maximum Correntropy Criterion Induced Losses for Regression. J. Mach. Learn. Res..

[3] He R., Zheng W.S., Hu B.G. (2011). Maximum Correntropy Criterion for Robust Face Recognition. IEEE Trans. Pattern Anal. Mach. Intell..

[4] Liu W.F., Pokharel P.P., Principe J.C. (2007). Correntropy: Properties and applications in non-Gaussian signal processing. IEEE Trans. Signal Process..

[5] Principe J.C. (2010). Renyi’s Entropy and Kernel Perspectives. Information Theoretic Learning.

[6] He R., Zheng W.S., Hu B.G., Kong X.W. (2011). A regularized correntropy framework for robust pattern recognition. Neural Comput..

[7] Bessa R.J., Miranda V., Gama J. (2009). Entropy and correntropy against minimum square error in offline and online three-day ahead wind power forecasting. IEEE Trans. Power Syst..

[8] He R., Hu B.G., Zheng W.S., Kong X.W. (2011). Robust Principal Component Analysis Based on Maximum Correntropy Criterion. IEEE Trans. Image Process..

[9] Chen B., Xing L., Liang J., Zheng N., Principe J.C. (2014). Steady-State Mean-Square Error Analysis for Adaptive Filtering under the Maximum Correntropy Criterion. IEEE Signal Process. Lett..

[10] Wu Z., Peng S., Chen B., Zhao H. (2015). Robust Hammerstein Adaptive Filtering under Maximum Correntropy Criterion. Entropy.

[11] Liu W., Pokharel P.P., Principe J.C. Error Entropy, Correntropy and M-Estimation. Proceedings of the 16th IEEE Signal Processing Society Workshop on Machine Learning for Signal Processing.

[12] Syed M.N., Pardalos P.M., Principe J.C. (2013). Invexity of the minimum error entropy criterion. IEEE Signal Process. Lett..

[13] Syed M.N., Pardalos P.M., Principe J.C. (2014). On the optimization properties of the correntropic loss function in data analysis. Optim. Lett..

[14] Marques de Sá J.P., Silva L.M.A., Santos J.M.F., Alexandre L.A. (2013). Minimum Error Entropy Classification.

[15] Cucker F., Zhou D.X. (2007). Learning Theory: An Approximation Theory Viewpoint.

[16] Singh A., Pokharel R., Principe J.C. (2014). The C-loss function for pattern classification. Pattern Recognit..

[17] Guo Z.C., Hu T., Shi L. (2018). Gradient descent for robust kernel-based regression. Inverse Prob..

[18] Smale F., Zhou D.X. (2007). Learning theory estimates via integral operators and their approximations. Constr. Approx..

[19] Lu S., Pereverzev S.V. (2013). Regularization Theory for Ill-Posed Problems: Selected Topics.

[20] Caponnetto A., Vito E.D. (2007). Optimal rates for the regularized least-squares algorithm. Found. Comput. Math..

[21] Steinwart I., Christmann A. (2008). Support Vector Machines.

[22] Bauer F., Pereverzev S.V., Rosasco L. (2007). On regularization algorithms in learning theory. J. Complexity.

[23] Feng Y.L., Fan J., Suykens J.A. (2017). A statistical learning approach to modal regression. arXiv.

[24] Ying Y., Pontil M. (2008). Online gradient descent learning algorithms. Found. Comput. Math..

